# Supplementary material for: A Humanized Yeast Phenomic Model of Deoxycytidine Kinase to Predict Genetic Buffering of Nucleoside Analog Cytotoxicity
Source: Genes (Basel). 2019 Sep 30;10(10):770. doi: 10.3390/genes10100770 (PMC6826991; doi:10.3390/genes10100770)
Supplement: Supplementary file 1 [file genes-10-00770-s001.zip › Additional_Files/Additional_File7_GOTermHeatmaps.zip!/Additional_File7_GOTermHeatmaps/mitochondrial_large_ribosomal_subunit.pdf]

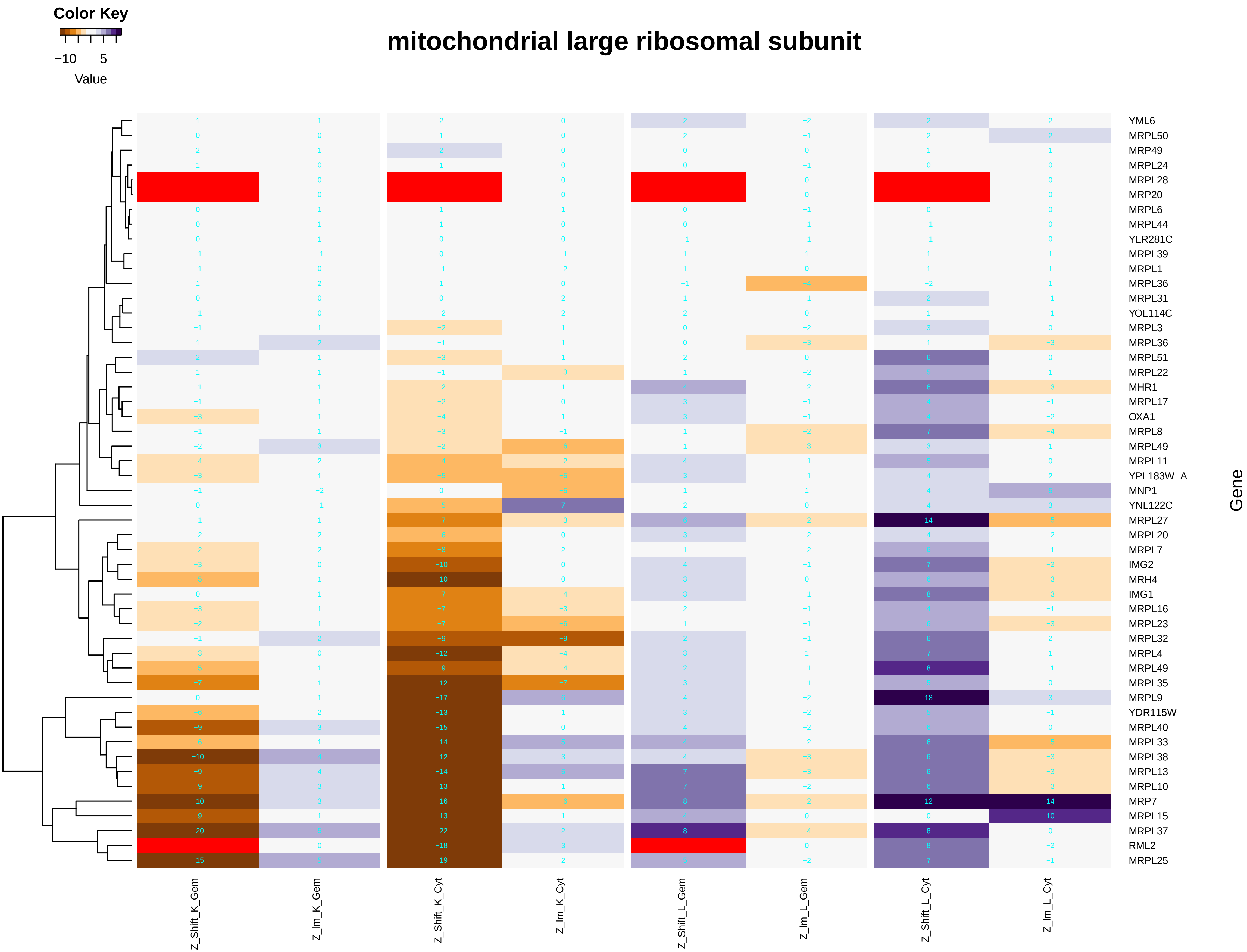
Color Key
−10
5
Value
mitochondrial large ribosomal subunit
YML6
MRPL50
MRP49
MRPL24
MRPL28
MRP20
MRPL6
MRPL44
YLR281C
MRPL39
MRPL1
MRPL36
MRPL31
YOL114C
MRPL3
MRPL36
MRPL51
MRPL22
MHR1
MRPL17
OXA1
MRPL8
MRPL49
MRPL11
YPL183W−A
MNP1
YNL122C
MRPL27
MRPL20
MRPL7
IMG2
MRH4
IMG1
MRPL16
MRPL23
MRPL32
MRPL4
MRPL49
MRPL35
MRPL9
YDR115W
MRPL40
MRPL33
MRPL38
MRPL13
MRPL10
MRP7
MRPL15
MRPL37
RML2
MRPL25
Gene
Z_Shift_K_Gem
Z_Im_K_Gem
Z_Shift_K_Cyt
Z_Im_K_Cyt
Z_Shift_L_Gem
Z_Im_L_Gem
Z_Shift_L_Cyt
Z_Im_L_Cyt
